# Cell Proliferation, Viability, Differentiation, and Apoptosis of Iron Oxide Labeled Stem Cells Transfected with Lipofectamine Assessed by MRI

**DOI:** 10.3390/jcm12062395

**Published:** 2023-03-20

**Authors:** Reza Jalli, Davood Mehrabani, Shahrokh Zare, Mahdi Saeedi Moghadam, Iman Jamhiri, Navid Manafi, Golshid Mehrabani, Janan Ghabanchi, Iman Razeghian Jahromi, Aghdass Rasouli-Nia, Feridoun Karimi-Busheri

**Affiliations:** 1 Medical Imaging Research Center, Shiraz University of Medical Sciences, Shiraz 71439-14693, Iran; 2 Stem Cell Technology Research Center, Shiraz University of Medical Sciences, Shiraz 71439-14693, Iran; 3 Burn and Wound Healing Research Center, Shiraz University of Medical Sciences, Shiraz 71439-14693, Iran; 4 Comparative and Experimental Medicine Center, Shiraz University of Medical Science, Shiraz 71439-14693, Iran; 5 Li Ka Shing Center for Health Research and Innovation, University of Alberta, Edmonton, AB T6G 1H9, Canada; 6 School of Medicine, Zanjan University of Medical Sciences, Zanjan 71439-14693, Iran; 7 School of Dentistry, Shiraz University of Medical Sciences, Shiraz 71439-14693, Iran; 8 Henry M. Goldman School of Dental Medicine, Boston University, Boston, MA 02215, USA; 9 Department of Oncology, Cross Cancer Institute, Faculty of Medicine, University of Alberta, Edmonton, AB T6G 1H9, Canada

**Keywords:** dental pulp stem cells, iron oxide nanoparticles, lipofectamine, tracking, MRI

## Abstract

To assess in vitro and in vivo tracking of iron oxide labeled stem cells transfected by lipofectamine using magnetic resonance imaging (MRI), rat dental pulp stem cells (DPSCs) were characterized, labeled with iron oxide nanoparticles, and then transfected with lipofectamine to facilitate the internalization of these nanoparticles. Cell proliferation, viability, differentiation, and apoptosis were investigated. Prussian blue staining and MRI were used to trace transfected labeled cells. DPSCs were a morphologically spindle shape, adherent to culture plates, and positive for adipogenic and osteogenic inductions. They expressed CD73 and CD90 markers and lacked CD34 and CD45. Iron oxide labeling and transfection with lipofectamine in DPSCs had no toxic impact on viability, proliferation, and differentiation, and did not induce any apoptosis. In vitro and in vivo internalization of iron oxide nanoparticles within DPSCs were confirmed by Prussian blue staining and MRI tracking. Prussian blue staining and MRI tracking in the absence of any toxic effects on cell viability, proliferation, differentiation, and apoptosis were safe and accurate to track DPSCs labeled with iron oxide and transfected with lipofectamine. MRI can be a useful imaging modality when treatment outcome is targeted.

## 1. Introduction

The transplantation of mesenchymal stem cells (MSCs) has been reported to be beneficial during organ shortage to restore normal organ function [1]. These cells possess strong bioactivity for modulating inflammation, enhancing angiogenesis, reducing tissue fibrosis, as well as immunosuppressive effects, multipotential differentiation abilities, self-renewal potential, and migratory properties, making them a candidate for clinical applications [2,3]. MSCs have successfully been used in regenerative medicine and isolated from various tissues including bone marrow [4], adipose [5], amniotic fluid [6], Wharton’s jelly [7], and dental pulp [8]. 

Among different types of MSCs, the dental pulp stem cells (DPSCs) that are located within a perivascular niche have strong proliferative and differentiation activities that make them a good candidate in regenerative medicine [9,10]. As in regenerative medicine, there has always been a great demand to track stem cells post-transplantation [11]; to help in tracing the labeled cells, various imaging methods have been applied after the transplantation of labeled cells [11]. Among imaging methods, magnetic resonance imaging (MRI) as a non-ionizing procedure has been utilized to track labeled stem cells after transplantation. It is a noninvasive method that can produce three-dimensional images with a high temporal and spatial resolution [11]. In MRI, contrast agents such as paramagnetic or supermagnetic metal ions have been recommended to induce special signal properties [12]. Among them, iron oxide nanoparticles can be imaged and easily traced by MRI. These particles have an iron oxide core diameter of 50–200 nm, are biocompatible and can be endocytosed into the cell [13], and can be successfully tracked by MRI when being endocytosed into stem cells [14]. They have minimal impact on cell function and cell phenotype too [15,16]. 

To facilitate the internalization of iron oxide nanoparticles, transfection materials have been recommended [17,18]. Among transfection materials, lipofectamine has been used as a nanocarrier to enable the encapsulation of nucleic acids into cells [19]. It is strongly biocompatible, which enhances delivery into cells, and is strongly applicable. In addition, lipofectamine is non-toxic, easily prepared, and can be produced on a large scale [20]. Lipofectamine can enable a high transfection across all nucleic acid types [21]. So, this study utilized lipofectamine to facilitate the internalization of iron oxide nanoparticles in rat DPSCs and their effects on cell proliferation, viability, and differentiation. The internalization of these particles has been evaluated by Prussian Blue staining and MRI tracking, both in vitro and in vivo. 

## 2. Materials and Methods

### 2.1. Isolation of DPSCs

Based on ethical issues to prepare human teeth and the distance and time interval to isolate DPSCs in a stem cell laboratory, 5 maxillae and mandibles of 5 male 4–6-week-old rats were used to isolate DPSCs from molar teeth [22,23], while the animals were euthanized under anesthesia utilizing 8 mg/kg of 2% xylazine (Alfasan, Woerden, The Netherland) and 50 mg/kg of 10% ketamine (Alfasan, The Netherland) [11]. The molar teeth could yield plenty of dental pulp to reach DPSCs easily, as described before [11], and even incisors were also reported to be rich in pulp [24,25]. The separated maxillae and mandibles were placed in a 50 mL falcon tube containing phosphate-buffered saline (PBS, Sigma, Urbank, CA, USA) and 3% penicillin streptomycin (Sigma, Urbank, CA, USA). In the maxillary and mandibular tissues of each animal, the connection between the bone and teeth were cut in an aseptic condition under a class I laminar flow hood, and the dental pulp tissues of all 5 rats were taken out from the molar teeth using a gauge 21 needle attached to a 1 mL syringe filled with Dulbecco’s Modified Eagle’s Medium F12 (DMEM-F12, Gibco, Waltham, MA, USA) [22]. 

The syringe content of all maxillary and mandibular tissues was later transferred to a 15 mL falcon tube containing DMEM-F12. Under a class II laminar flow hood, the dental pulp tissues were washed three times in PBS and then, these intact pulp tissues were chopped into small pieces by a sterile blade, and transferred into a 15 mL falcon tube containing 5 mL of DMEM-F12. The falcon content was later centrifuged at 200× *g* for 10 min, the supernatant was taken out, and the remaining content was treated with 1.5 mL of 0.14% collagenase type I (Gibco, Waltham, MA, USA) for 45 min. It was later transferred to an incubator at 37 °C with 5% CO_2_ and saturated humidity. Then, 5 mL of DMEM-F12 and 10% fetal bovine serum (FBS, Gibco, Waltham, MA, USA) were added to a falcon tube, and the tube was spun at 200× *g* for 7 min. The supernatant was removed, and the remaining contents were re-suspended in 1 mL of media containing 10% fetal bovine serum, 1% non-essential amino acids (Sigma, USA), 1% penicillin streptomycin, and 88% DMEM-F12 [10]. 

The suspended cells were transferred into a culture flask containing 4 mL of DMEM-F12, 1% penicillin streptomycin, 1% non-essential amino acids, and 10% fetal bovine serum, and the culture flask was placed in an incubator at 37 °C with 5% CO_2_ and saturated humidity. The media were changed every 3 days to reach 80% confluence; while subculturing of cells was performed at 80% confluence by treating the cells with 0.25% *w*/*v* trypsin-EDTA (Gibco, Waltham, MA, USA) until the 3^rd^ passage. Meanwhile, the cells were also evaluated under an invert microscope (Nikon, Tokyo, Japan) for their morphology, and pictures were prepared by a digital camera (Olympus, Tokyo, Japan) at all passages [23]. The Ethics Committee of the National Institute for Medical Research Development of Iran Ministry of Health, Treatment and Medical Education (Ethical code: 963474) approved all experimental protocols. Animal experiments were performed based on Iran Veterinary Organization guidelines and according to Preferred Reporting Items for Animal studies in Endodontology (PRIASE) 2021 guidelines [26].

### 2.2. Cell Characterization

To characterize DPSCs, they were assessed morphologically at passages 1–3 to be a spindle shape by an invert microscope. For the osteogenic differentiation property, DPSCs were transferred into a 6-well plate (Corning, Corning, NY, USA) which consisted of complete culture media to reach 80% confluence, while the osteogenic media were added and consisted of complete culture media supplemented with 100 nM dexamethasone (Sigma, USA), 50 µM ascorbic acid (Merck, Darmstadt, Germany), 10 mM glycerol 3-phosohate (Merck, Darmstadt, Germany), and 15% fetal bovine serum for 21 days. The modification of media happened every 2 days and, after 21 days, the cells were treated with 10% formalin for 20 min to be fixed. Then, several washings with deionized water were carried out, and, later, cells were stained with fresh Alizarin Red solution solved in double distilled water (DDW) at a pH of 4.1 (Sigma, USA) to detect calcium deposits in red color, verifying an osteogenic induction. To investigate adipogenic differentiation, DPSCs were transferred into a 6-well plate which consisted of complete culture medium to reach 80% confluence. The changing of the media was conducted with adipogenic medium, which contained a complete culture medium supplemented with 100 nM dexamethasone, 100 µM ascorbic acid, 200 μM of indomethacin (Sigma, Urbank, CA, USA), and 15% fetal bovine serum, for 21 days. The 10% buffered formalin solution was treated with cells for 20 min to fix the cells, and, after several washings with deionized water, the cells were stained with fresh 0.5% Oil Red-O (Sigma, USA) dissolved in 2-propanol solution (Merck, Germany) for 2 h, and this verified adipogenic induction by red droplets [27,28,29].

Reverse transcriptase–polymerase chain reaction (RT–PCR) was used to determine the expression of 208 bp CD73, and 177 bp CD90 as mesenchymal markers, and 257 bp CD34, and 450 bp CD45 as hematopoietic markers. To reach the total RNA, an RNA extraction kit (Cinna Gen, Tehran, Iran) was used based on the manufacturer’s protocol. The first-strand cDNA was prepared applying the Revert Aid™ first strand cDNA synthesis kit (Thermo Fisher, Waltham, MA, USA). PCR thermal cycler (Veriti Thermal Cycler, Foster City, CA, USA) was used for PCR runs, including 1 cycle at 94 °C for 3 min, 35 cycles at 94 °C for 30 s, 60 °C for 30 s, and 72 °C for 30 s, and 1 cycle at 72 °C for 10 min, and, finally, the bands were visualized by electrophoresis [30] (Table 1).

### 2.3. Growth Kinetics

The trypan blue exclusion test was used to determine the growth kinetics and the cell viability of DPSCs at the 3rd passage, labeled with 180 µg/mL iron oxide [Iron (II, III) oxide nano powder, 50–100 nm particle size (SEM), 97% trace metals basis, Sigma-Aldrich 637106, Milwaukee, WI, USA] and transfected with 1 µL/mL lipofectamine while the cells were cultured in 24-well plates for 6 days at 22,000 cells/well (Corning, Corning, NY, USA). The 0.4% trypan blue solution (Sigma, Milwaukee, WI, USA) was added to the cell suspension, and later the cells were counted under a phase contrast microscope (Olympus, FSX100, Tokyo, Japan) using a Neubauer hemocytometer slide. The population doubling time (PDT) in an hour and the growth curve were determined until 6 days by the formula below:

$$\mathrm{PDT}=T\times \frac{ln2}{\ln\frac{Xe}{Xb}}\;\mathrm{and}\;N=\frac{n1+n2+n3+n4}{4}\times 2\times v\times 1000$$

While T is the incubation time in h, Xb is the cell number at the beginning of the incubation time and Xe is the cell number at the end of the incubation time, Ln = log_e_ and e = Neperian number. 

### 2.4. Cell Labeling

Different concentrations of iron oxide [90, 180 and 260 μg/mL, 50–100 nm particle size (SEM), 97% trace metals basis] were prepared and utilized for cell labeling, and further cell tracking. Lipofectamine was used for transfection of DPSCs to facilitate the internalization of iron oxide. In summary, DPSCs at the 3rd passage were cultured using just DMEM-F12 in the absence of any FBS and penicillin–streptomycin. Later, media change was conducted using an equal amount of DMEM-F12 containing 1 or 2 μL/mL of lipofectamine 2000 (L2000; Invitrogen, Carlsbad, CA, USA) at room temperature for 15 min, and then was transferred to an incubator at 37 °C with 5% Co_2_ and saturated humidity [31]. 

After removing the media, the cells in culture flasks were washed three times with PBS, trypsinized, re-washed with PBS three times, and finally centrifuged at 200× *g* for 5 min. The supernatant was discarded, and the cell pellet was washed three times with PBS again. The cell pellet was later suspended in 200 µL of PBS and 200 µL of 15% agarose gel (Sigma, Milwaukee, WI, USA) in 1.5 mL tubes. The tubes were grouped for MRI as follows: Group 1 just contained 15% agarose gel. In groups 2–6, 180, 90, 70, 50, and 30 µg/mL of iron oxide were added for cell labeling, respectively; while in groups 2–6, 1 μL/mL of lipofectamine was also used for transfection of DPSCs together with 15% agarose gel. Group 7 was non-coated iron oxide nanoparticles, and group 8 consisted of just H_2_O. 

### 2.5. The 3-4,5-Dimethylthiazol-2-yl)-2,5-diphenyl-2H-tetrazolium Bromide (MTT) Assay

As the 3-4,5-dimethylthiazol-2-yl)-2,5-diphenyl-2H-tetrazolium bromide (MTT) assay provides information about mitochondrial respiratory chain activity and the mitochondrial oxidation state, regarding the effect of labeling with iron oxide at various concentrations and transfection with lipofectamine on cell proliferation at the 3rd passage, the cells were plated in 96-well plates at 5000 cells/200 µL (Corning, Corning, NY, USA); meanwhile, the media change happened after 24 h with (i) just culture media (control group), or (ii) iron oxide nanoparticles at various concentrations (90, 180, and 260 μg/mL) transfected with lipofectamine (1 or 2 μL/mL) in culture media (experimental group). After 24 h, 20 µL of a solution consisted of 5 mg/mL of dimethylthiazol-2-yl)-2,5-diphenyltetrazolium bromide, and a tetrazole (Sigma, Milwaukee, WI, USA) was added to each well and transferred to an incubator at 37 °C with 5% CO_2_ and saturated humidity for 4 h. The removal of media was gently undertaken and to dissolve the formazan crystals, 200 µL of DMSO/well (Merck, Germany) was later added. Cell viability was investigated and repeated 4 times on a microplate reader (Floustar Omega, BMG LabTech, Ortenberg, Germany) at an optical density of 570 nm. The defined following formula was used to determine the cell viability, while Ac and Ab were considered as the absorbance in control and the blank wells.

$$Survival\;rate(\%)=\frac{A\;sample-Ab}{Ac-Ab}\times 100$$

### 2.6. In Vitro Tracking by MRI

A clinical 1.5 T MRI scanner (General Electric, Signa HDXt, 1.5 T, USA) was used for in vivo and in vitro imaging. The case and control rats were placed in a head coil. T2* gradient echo pulse sequence was utilized with a flip angle of 25°, echo time of 24 ms, repetition time of 720 ms, field of view of 250 × 250, and matrix size of 288 × 160. The phantom was a polyethylene tube. Tube 1 just contained 15% agarose gel, Tubes 2 to 6 had 1 µL/mL of lipofectamine and 15% agarose gel for transfection of DPSCs, together with 180, 90, 70, 50 and 30 µg/mL of iron oxide for cell labeling, respectively. Tube 7 consisted of just water (H_2_O). The phantom was placed in a head coil for imaging using a T2* pulse sequence, slice thickness of 3 mm, repetition time of 460 ms, echo time of 24 ms, magnetic field strength of 1.5 T, flip angle of 25°, matrix size of 512 × 512, and field of view of 210 × 210 [11]. 

As iron oxide nanoparticles had a small crystal size, the orientation of the lattice did not hinder the magnetic moments. They significantly shortened the T2 relaxation time leading to the creation of a low signal contrast on spin-echo sequences. On T2* gradient echo images, among different concentrations of iron oxide, the concentrations of 180 μg/mL and 1 µ/mL lipofectamine were used for cell labeling and transfection, respectively. The SNR of T2* weighted images were inherently lower than T2 images. The higher value was the concentration of iron oxide; it resulted in a more hypo-intense signal and larger diameter blooming artifacts. Regarding the background, the image of each cell was cropped which resulted in a white background. In this study, the signal at different points of time was not determined.

### 2.7. In Vivo Tracking by MRI

Cells at the 3rd passage, labeled with 180 μg/mL of iron oxide nanoparticles and transfected with 1 µL/mL of lipofectamine, were used for transfection and in vivo tracking of the internalization of iron oxide nanoparticles. In summary, they were trypsinized and centrifuged at 200× *g* for 5 min. The supernatant was removed, and the cell pellet was washed three times with PBS. The iron oxide labeled cells were later injected intraperitoneally in two adult rats. Then, in vivo MRI was undertaken for the 2 recipient animals using the same clinical 1.5 T MRI scanner utilized in vitro. The rats were randomly allocated to experimental (*n* = 2) and control groups (*n* = 2) for MRI analysis, and in vivo tracing of iron oxide labeled cells transfected with lipofectamine. It is necessary to mention that a 1.5 mL tube with identical volumes was utilized for all groups. The cells were suspended in 200 µL of PBS and 200 µL 15% agarose gel (Sigma, Milwaukee, WI, USA). 

### 2.8. Prussian Blue (PB) Staining

Prussian Blue staining (Polysciences, Warrington, PA, USA) was conducted to visualize intracellular iron content and localization to assess the in vitro efficiency of the uptake for 180 μg/mL iron oxide when transfected with 1 µL/mL of lipofectamine to facilitate the internalization of iron oxide nanoparticles. After labeling, cells were fixed in 4% glutaraldehyde for 10 min and washed in PBS. Staining included two changes of 2% potassium ferrocyanide (Sigma, Milwaukee, WI, USA) in 2% hydrochloric acid solution (Merck, Germany) at 10 min each. Cells were washed in PBS, and pictures were prepared of representative fields using an upright, light microscope (Olympus, FSX100, Tokyo, Japan) and digital camera (QImaging, Surrey, BC, Canada), while iron oxide nanoparticles were seen in bright blue, nuclei in red, and cytoplasm in pink color [31]. 

### 2.9. Bax and Bcl-2 Gene Expression Assay

For Bax and Bcl-2 gene expression assay, an RNA extraction kit (Cinna Gen, Tehran, Iran) was used to recover the total cellular RNA from DPSCs labeled with 180 μg/mL of iron oxide and transfected with 1 µL/mL of lipofectamine. To assess the quantity and quality of obtained RNA, the ratio of optical density (A_260_/A_280_ and A_260_/A_230_) was measured, and the concentration was determined by a Nanodrop™ spectrophotometer (Nanodrop, Thermo Fisher, Waltham, MA, USA). They were kept at −80 °C until further cDNA synthesis. The cDNA was prepared using 1000 ng total RNA in a first-strand cDNA synthesis reaction applying a Revert Aid™ first strand cDNA synthesis kit (Thermo Fisher, Waltham, MA, USA). Bax and Bcl-2 genes were used as targets and Beta-2-Microglobulin (B2m) as an endogenous housekeeper control. The sequences of targeted genes were prepared using NCBI database, and primer software 3 was used to design the primer sets. Oligonucleotide sequences for 244 bp B2m, 134 bp Bcl-2, and 174 bp Bax were illustrated in Table 2.

Real-time PCR was used, applying the SYBR Green I as reporter dye and Step One Real Time PCR reactions (Applied Biosystems, San Francisco, CA, USA) on the third day. The RealQ Plus 2× Master Mix (Green Ampliqon A/S, Odense, Denmark) was used, and in each reaction, 200 nM of each primer was added to target the specific sequence. The qPCR condition was as follows: 10 min at 94 °C, 40 cycles of 15 sec at 94 °C, 60 sec at 60 °C, and the melting curve analysis ramped up from 65 °C to 95 °C. The amplification signals of different samples were normalized to the B2m cycle threshold (Ct), and the 2^−ΔΔCt^ method compared the mRNA level for cell groups, which was represented as fold-change in data analysis. The Bax/Bcl-2 ratio was evaluated too. 

### 2.10. Apoptosis Analysis by Flow Cytometry

In total, 1 × 10^6^ DPSCs were labeled with 180 μg/mL of iron oxide and transfected with 1 µL/mL of lipofectamine to determine apoptosis by flow cytometry. In brief, they were trypsinized and transferred into a falcon tube. Using a cold PBS solution, the cells were centrifuged at 200× *g* for 5 min. The supernatant was removed, and the cell pellet was washed two times with Annexin V-PE early apoptosis detection kit I (BD Biosciences, Billerica, MA, USA) suspended in 0.1 mL of Annexin V binding buffer 1X (Invitrogen, USA). Then, addition of 5 µL of Annexin V-PE (Invitrogen, Carlsbad, CA, USA) and 5 μL of 7-Aminoactinomycin D 7-AAD (Invitrogen, USA) took place. They were slowly vortexed and then left for 15 min at room temperature. In total, 400 μL of Annexin V binding buffer (1×) was added as attachment buffer and was further read by a Fluorescence-Activated Cell Sorting (FACS) Calibur flow cytometer (BD Biosciences, Germany) for an hour, and was finally analyzed by the Flow Jow software. The amounts of early apoptosis were determined as the population percentage of Annexin V+/7-AAD, and late apoptosis as the population percentage of Annexin V+/7-AAD + labeled cells with 180 μg/mL of iron oxide and transfected with 1 µ/mL of lipofectamine. 

### 2.11. Statistical Analysis

Prism software (Graph Pad Software Inc., version 6.0, San Diego, CA, USA) was used for the processing and managing of data. Prism is specifically designed to analyze the data we wanted to run, such as by providing an analysis of quantitative and categorical data to make it easier to enter data properly, choose suitable analyses, and create stunning graphs. The groups were compared by one-way analysis of variance (ANOVA). A *p* value less than 0.05 was considered statistically significant.

### 2.12. Ethics Approval

The study was performed according to the good clinical practices recommended by the Declaration of Helsinki and its amendments. Local ethical approval was obtained too (Ethical code: 963474). 

## 3. Results

### 3.1. Cell Characterization

**Figure 1:**
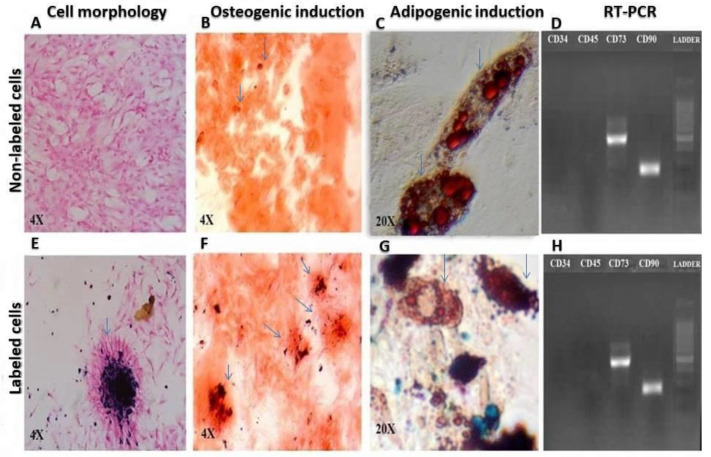
Comparison of cell morphology: (**A**) Non-labeled and (**E**) Lipofectamine-transfected iron oxide labeled cells, pointed by arrow, 4×, osteogenic induction; (**B**) Non-labeled and (**F**) Lipofectamine-transfected iron oxide nanoparticles labeled cells, pointed by arrow, 4×, adipogenic differentiation; (**C**) Non-labeled and (**G**) Lipofectamine-transfected iron oxide labeled cells, pointed by arrow, 20× and RT-PCR for expression of mesenchymal and hematopoietic markers; (**D**) Non-labeled and (**H**) Lipofectamine-transfected iron oxide labeled cells).

Both labeled cells with 180 μg/mL of iron oxide transfected with 1 µL/mL of lipofectamine and non-labeled DPSCs were spindle-shaped and adherent to the culture plates (Figure 1A,E: 4×). In osteogenic induction, lipofectamine-transfected labeled and non-labeled cells revealed intracellular calcium deposits in red color after staining with Alizarin Red (Figure 1B,F: 4×). In adipogenic induction, lipofectamine-transfected labeled and non-labeled cells showed intracellular lipid droplets in red color when stained with Oil Red O (Figure 1C,G: 20×). The RT–PCR of lipofectamine-transfected labeled and non-labeled cells denoted to the positive expression of CD73 and CD90 as mesenchymal markers, and negative expression of CD34 and CD45 as hematopoietic markers (Figure 1D,H).

### 3.2. MTT Assay

**Figure 2:**
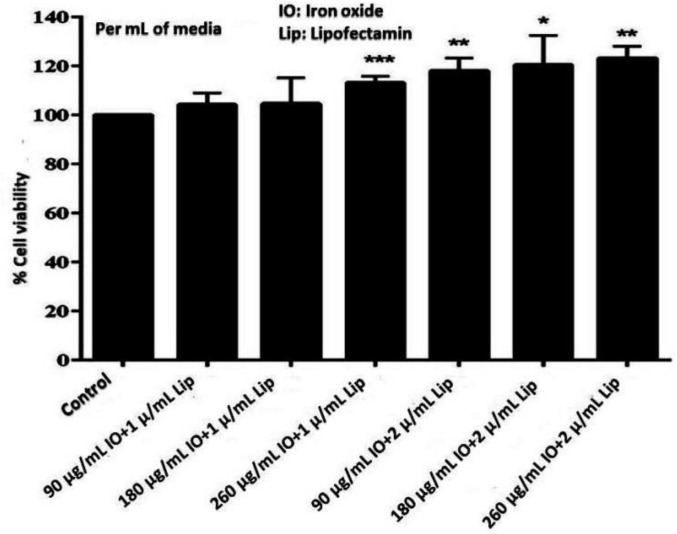
MTT assay regarding cell proliferation and viability of labeled cells in different concentration of iron oxide nanoparticles (90, 180, 260 µg/mL) transfected with 1 and 2 µL/mL of lipofectamine. Data are representative of three independent experiments (*p* = 0.19, * *p* = 0.048, ** *p* = 0.001, *** *p* = 0.003, respectively), revealing the non-toxic role of nanoparticles when transfected with lipofectamine.

The MTT assay did not reveal any significant decrease in cell proliferation and viability when DPSCs were treated with different concentrations of iron oxide nanoparticles (90, 180, 260 µg/mL) and transfected with lipofectamine (1 and 2 µL/mL) in the culture media in comparison to the control of non-labeled cells, thus revealing the non-toxic role of nanoparticles when transfected with lipofectamine. Data are representative of three independent experiments (*p* = 0.19, * *p* = 0.048, ** *p* = 0.001, *** *p* = 0.003, respectively, Figure 2).

### 3.3. Growth Kinetics

**Figure 3:**
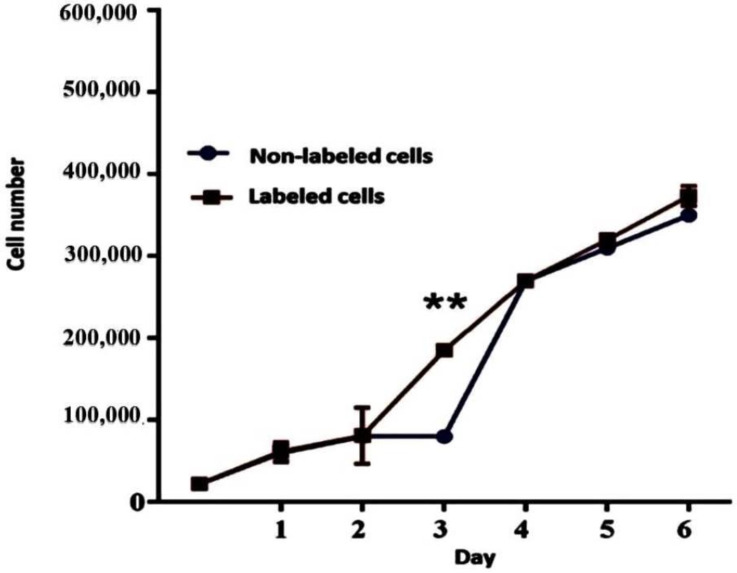
The population doubling time (PDT) in an hour and the growth curve were determined until day 6. The number of cells treated with 180 µg/mL of iron oxide transfected with 1 µL/mL of lipofectamine (Day 1: *p =* 0.92, day 2: *p =* 0.97, day 3: ** *p =* 0.0001, day 4: *p =* 0.95, day 5: *p =* 0.28, day 6: *p =* 0.155), revealing the PDT of 36 h and 33 h for non-labeled and iron oxide labeled cells (180 µg/mL) transfected with 1 µL/mL lipofectamine, respectively.

The number of cells for non-labeled and 180 µg/mL of iron oxide labeled cells transfected with 1 µL/mL lipofectamine for 6 days was presented in Figure 3, revealing the PDT of 36 h and 33 h for non-labeled and iron oxide labeled cells (180 µg/mL) transfected with 1 µL/mL lipofectamine, respectively. The findings reveal that 180 µg/mL of iron oxide labeled cells transfected with 1 µL/mL lipofectamine had a significant proliferation rate in comparison with non-labeled cells, denoting to the non-toxic impact of nanoparticles transfected with lipofectamine (Control group) (Day 1: *p =* 0.92, day 2: *p =* 0.97, day 3: ** *p =* 0.0001, day 4: *p =* 0.95, day 5: *p =* 0.28, day 6: *p =* 0.155).

### 3.4. In Vitro MRI

**Figure 4:**
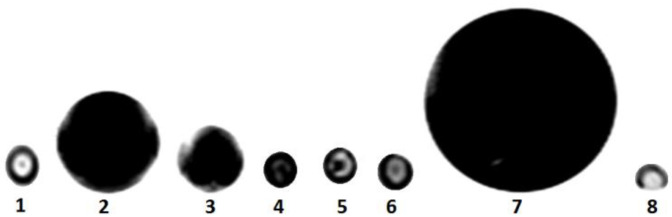
The T2* weighted MR imaging of in vitro samples. Group 1 contained 15% agarose gel only. In groups 2–6, 1 µL/mL of lipofectamine and 15% agarose gel were used for transfection of DPSCs and then 180, 90, 70, 50, and 30 µg/mL of iron oxide were applied for cell labeling, respectively. Group 7 was non-coated iron oxide nanoparticles and group 8 consisted of just H_2_O.

Figure 4 shows the T2* weighted MR image of in vitro samples, denoting an increase in hypointensity when cells were treated with 180 µg/mL of iron oxide and transfected with 1 µL/mL of lipofectamine. T2* was demonstrated to be shorter due to higher numbers of iron oxide in labeled cells transfected with 1 µL/mL of lipofectamine. In vitro MRI detection thresholds were set at a T2* value of 0.084 ms when DPSCs were labeled with 180 µg/mL of iron oxide and transfected with 1 µL/mL of lipofectamine. The T2* had less than the anticipated value (<5 ms). A greater signal loss was observed over a greater area in non-labeled cells when tracked by MRI.

### 3.5. In Vivo MRI

**Figure 5:**
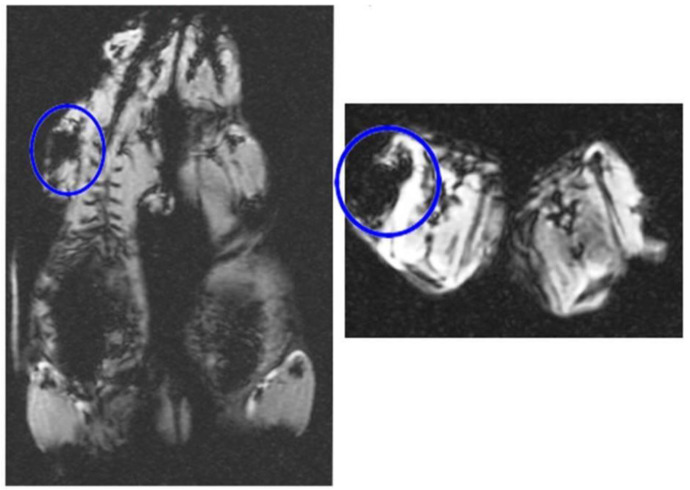
The T2* weighted images of in vivo samples showing the hypo-intense signal of 180 µg/mL of iron oxide labeled DPSCs transfected with 1 µL/mL lipofectamine that confirms presence of iron oxide in the labeled cells in comparison to in vivo samples of non-labeled cells showing lack of this MRI appearance (Left and Right).

Figure 5 in in vivo MRI tracking after intraperitoneal injection of DPSCs labeled with 180 µg/mL of iron oxide transfected with 1 µL/mL lipofectamine confirmed presence of iron oxide in the labeled cells transfected with lipofectamine in the peritoneal cavity. The T2* weighted images of in vivo samples in the right picture displays the hypo-intense signal of the iron oxide contrast compared to non-labeled cells in the left picture lacking this MRI appearance.

### 3.6. Prussian Blue Staining

**Figure 6:**
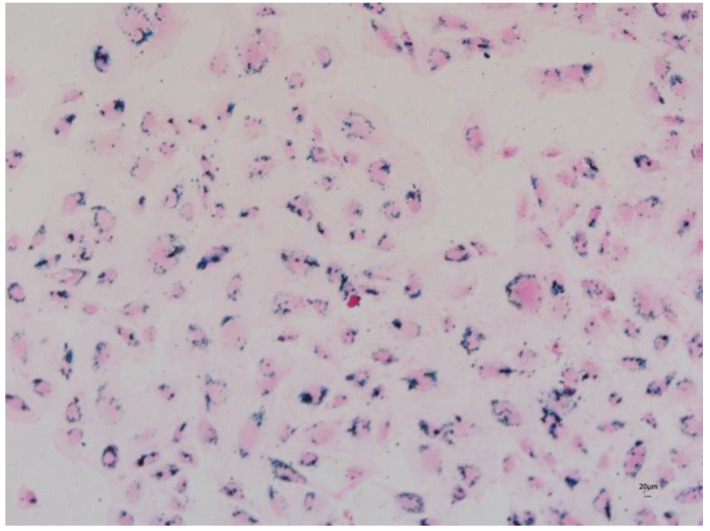
Prussian Blue staining of DPSCs labeled with 180 µg/mL of iron oxide and transfected with 1 µL/mL of lipofectamine to facilitate internalization of iron oxide nanoparticles that appear in blue color (4×).

The passive incubation period of labeled cells with 180 μg/mL of iron oxide and transfection with 1 µL/mL of lipofectamine showed internalization of the particles within DPSCs that looked blue after being stained with Prussian Blue. The blue color was marked with an arrow (Figure 6).

### 3.7. Bax and Bcl-2 Gene Expression

**Figure 7:**
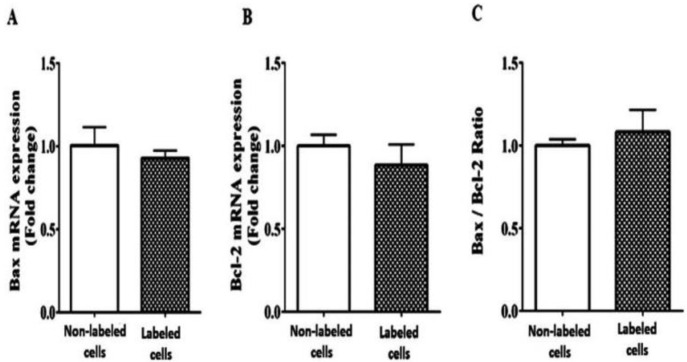
The effect of 180 µg/mL of iron oxide transfected with 1 µL/mL of lipofectamine on gene expression of Bax pro-apoptotic gene (**A**), Bcl-2 anti-apoptotic genes (**B**), and Bax/Bcl-2 ratio (**C**) of labeled DPSCs (Bcl-2: *p* = 0.41_,_ Bax: *p* = 0.38, and Bax/Bcl-2 ratio: *p* = 0.58), revealing that 180 µg/mL of iron oxide transfected with 1 µL/mL lipofectamine in labeled DPSCs did not induce any significant apoptosis when used in cell labeling.

Real time PCR findings denoted the gene expression of the anti-apoptotic gene of Bcl-2 as 0.92-fold when cells were labeled with 180 µg/mL iron oxide and transfected with 1 µL/mL lipofectamine in comparison to non-labeled cells. The gene expression of pro-apoptotic gene Bax in cells labeled with 180 µg/mL iron oxide and transfected with 1 µL/mL lipofectamine was 0.88-fold when compared with non-labeled cells. The ratio of Bax to Bcl-2 (Bax/Bcl-2) for labeled cells transfected with lipofectamine was 1.04-fold when compared to non-labeled cells. No significant difference was noticed between labeled and non-labeled cells for the gene expression of Bcl-2 (*p* = 0.41), Bax (*p* = 0.38), and Bax/Bcl-2 ratio (*p* = 0.58, Figure 7), revealing that 180 µg/mL of iron oxide transfected with 1 µL/mL lipofectamine in labeled DPSCs did not induce any significant apoptosis when used in cell labeling.

### 3.8. Apoptosis Analysis by Flow Cytometry

**Figure 8:**
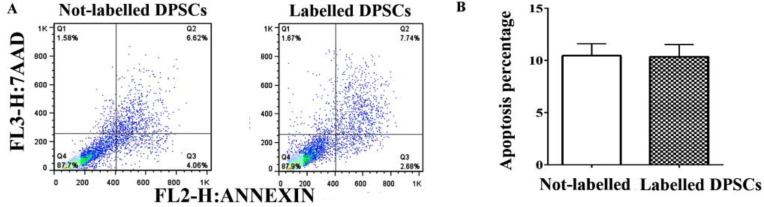
The flow cytometry analysis of annexin V+/7-AAD+ on DPSCs labeled with 180 μg/mL of iron oxide transfected with 1 µL/mL of lipofectamine. (**A**) Dead cells were scored as necrotic annexin V: negative/7-AAD: positive, upper left quadrants, Q1, or late apoptotic annexin V-positive/7-AAD-positive, upper right quadrants, Q2, early apoptotic annexin V-positive/7-AAD-negative, lower right quadrants, Q3, and following a gating on the normal cells that were considered viable were PE Annexin V and 7-AAD negative lower left quadrants, Q4. (**B**) The percentage of early and late apoptosis was indicated in a bar chart. Data presented were indicative of three independent experiments (*p* = 0.95).

As Figure 8 demonstrates, DPSCs that were negative for PE Annexin V and 7-AAD were at the starting point of the apoptotic process. When they were positive for PE-Annexin V and 7-AAD, they were in final stage of the apoptotic process and headed towards cell death. The cells that were negatively affected by PE Annexin V and 7-AAD were identified as viable cells. The flow cytometry findings regarding the analysis of annexin V+/7-AAD+ on DPSCs labeled with 180 μg/mL of iron oxide and transfected with 1 µL/mL of lipofectamine showed a 0.5% decrease in the apoptotic process (*p* = 0.95).

## 4. Discussion

DPSCs with self-renewing, proliferation, and differentiation properties can be a favorable substitute for tissue repair [8,9,11]. They have been used in regenerative medicine [32], including pulp regeneration [33], oral maxillofacial tissue engineering [34], and many other diseases [35], but there are still limitations in their clinical application. Therefore, an effective method to be non-invasive and to accurately track the survival and migration of these grafted cells can open a door in cell transplantation. To track the cells, currently different procedures have been used, including cell labeling with iron oxide nanoparticles [36]. Iron oxide nanoparticles are contrast magnetic examples that can be traced by MRI with a resolution of 25 to 50 microns that can approach the resolution of a single cell [11,31]. 

In MRI, iron oxide nanoparticles, by shortening the nuclear magnetic resonance T2 relaxation time over and far beyond the usual dipole–dipole relaxation mechanism, impact both T1 and T2 relaxation time, and, due to the predominant T2 effect, these agents usually create a hypointense contrast with conventional spin-echo MR sequences. So, MRI seems to be a feasible and ideal approach to trace the biodistribution and migration of labeled stem cells after cell transplantation [11,26]. In our study, we have labeled DPSCs with 180 µg/mL of iron oxide and transfected them with 1 µL/mL of lipofectamine and have shown that these nanoparticles were internalized in the cytoplasm by MRI and Prussian Blue staining methods. Iron oxide nanoparticles and lipofectamine were demonstrated not to interfere with the proliferation and differentiation capacities of DPSCs, and did not induce any cell apoptosis. The safe internalization of iron nanoparticles into stem cells through the endocytotic pathway has been described before [37,38,39]. 

Cardarelli et al. demonstrated that Brownian diffusion is an efficient route for lipofectamine/DNA complexes to be utilized for optimal transfection and to avoid metabolic degradation. It could improve the intracellular trafficking characterization [40]. The important role of lipofectamine as a nanocarrier to enable encapsulation of nucleic acids into cells [19] and to enhance the delivery of nanoparticles into cells was described before [20]. Caglayan et al. found that elevated concentrations of mirVana or locked nucleic acid miR-15a-5p mimic that were delivered to endothelial cells using lipofectamine RNAiMAX as a carrier specifically increased the expression of miR-15a-5p [41]. Magro et al. revealed that nanomaterials can be successfully internalized into mesenchymal stem cells transfected with lipofectamine by an increase in GFP expression [42]. These findings are in line with our results that the transfection of iron oxide with lipofectamine in labeled stem cells could potentiate the internalization of iron nanoparticles in the absence of any toxicity for cell proliferation and differentiation. 

It is also crucial to evaluate the susceptibility of labeled cells to apoptosis as transplanted labeled cells may encounter oxidative stress, radical formation, inflammation, and physical alterations. It has been mentioned that the Fe^2+^ ion can react with hydrogen peroxide and produce the hydroxyl radical that can cause tissue and DNA damages [43]. Our findings with MTT assay revealed that cell labeling with 180 μg/mL of iron oxide and transfection with 1 µL/mL of lipofectamine had no negative effect on cell viability [44]. Our group, in their previous study, did not reveal any reduction in proliferation of labeled stem cells with iron oxide nanoparticles too [45].

The detection of iron oxide nanoparticles in labeled cells by MRI can be due to the presence of inert magnetite (Fe_3_O_4_) or maghemite (γ-Fe_2_O_3_) iron core [33]. Fe_3_O_4_ or γ-Fe_2_O_3_ iron cores were also shown to have little or no negative effect on cell proliferation and viability which can explain our results on the absence of any toxicity on cell proliferation, viability, and differentiation [36]. In a previous report by our group using iron oxide nanoparticles coated with 3.5 mg/mL dextran, further MRI tracking and Prussian Blue staining could reveal the presence of iron oxide, even in cells where no lipofectamine was applied for iron oxide internalization. It seems that the addition of 1 µL/mL of lipofectamine in the present study to iron oxide labeled cells did not induce any cell toxicity either. No toxic effects on cell proliferation, viability, and differentiation were reported before [11], similar to our present study. Differentiation properties of labeled stem cells have not been affected in the previous works [11,45,46], which is in line with our present work. 

Recognizing the verge of apoptosis in labeled cells with iron oxide particles is of great importance in regenerative medicine, so assessment of Bax/Bcl-2 ratio can have a vital role in determining the cell proliferation and viability [11]. Our results presented a lack of any apoptosis when DPSCs were labeled with iron oxide and transfected with lipofectamine; this is in agreement with reports demonstrating that magnetic nanoparticles taken up by stem cells did not induce any apoptosis [11,19,47,48]. 

In our study, 180 μg/mL iron oxide nanoparticles transfected with 1 µL/mL of lipofectamine were applied in cell labeling. They were successfully visualized by MRI when applying T2* weighted images. The high relaxivity and sensitivity of MRI affecting homogeneity of the local magnetic field and the production of a large paramagnetism could reveal the presence of iron oxide nanoparticles with hypo-intense signals [11,31,49,50,51]. Our study, where cells were labeled with iron oxide and transfected with lipofectamine, similarly showed the largest signal intensity on T2WI, and, in contrast, T1 weighted images were weak in detecting iron oxide in labeled cells [11,31,52]. Similarly, our group, in previous research on T2W images of injected rats with iron oxide labeled cells, indicated the negative contrast of iron oxide nanoparticles, and the absence of any sign of negative contrast in axial images, where no iron oxide was present [44]. Lin et al. presented a damping signal in the transplanted area of cells labeled with iron oxide and transfected with lipofectamine on both T2 weighted and T2* gradient-echo images, revealing hypointense signals for the identification of grafted cells. In grafted cells labeled with iron oxide, no toxic effects have been previously reported for cell proliferation and viability [31]. Fast dephasing in adjacent protons, and the decrease in transverse (T2) and translational (T1) relaxation times were also mentioned to affect the iron oxide crystals and the nearby protons [53], while a shorter transverse relaxation time can produce a darker image around the iron oxide nanoparticles, and was considered as a negative contrast [54]. In our study, 1.5 T MRI has been applied for tracing labeled stem cells, and to reach the best contrast to visualize the labeled cells. Similarly, three T MRI scanners have been used to detect iron oxide in labeled cells [55]. An increased retention of the magnetic nanoparticles in labeled cells can be beneficial over a longer period of time in tracing iron oxide labeled cells. As the contrast agent is diluted and/or removed during each cell cycle, the longevity of the detectable MRI signal is reduced. In addition, the transfection method can influence the rate of removal of the contrast agent during cell cycling [43]. 

## 5. Limitations

The limitations that existed in our study were MRI tracking in rats, which was not available in our area for laboratory animals, and also MRI, which was not easily affordable, except in several practices by an expert radiologist.

## 6. Conclusions

In conclusion, it was illustrated that the transfection of iron oxide labeled stem cells with lipofectamine could facilitate the internalization of iron oxide within cells, in the absence of any toxic effects on cell proliferation, viability, differentiation, and susceptibility to apoptosis. The intracellular distribution of iron oxide was documented by Prussian Blue staining and MRI tracking. MRI was shown to be a safe, non-invasive imaging method to track labeled stem cells. It seems that MRI can be a useful modality in the assessment of treatment outcome when labeled stem cells are transplanted in clinical practice. 

## Figures and Tables

**Table 1:** The described CD73, CD90, CD34, and CD45 sequences.

Gene	Primer Sequence	Size (bp)
CD34	Forward: 5′-AGCCATGTGCTCACACATCA-3′	257
	Reverse: 5′-CAAACACTCGGGCCTAACCT-3′	
CD45	Forward: 5′-CCAAGAGTGGCTCAGAAGGG-3′	450
	Reverse: 5′-CTGGGCTCATGGGACCATTT-3′	
CD73	Forward: 5′-TGCATCGATATGGCCAGTCC-3′	208
	Reverse: 5′-AATCCATCCCCACCGTTGAC-3′	
CD90	Forward: 5′-GACCCAGGACGGAGCTATTG-3′	177
	Reverse: 5′-TCATGCTGGATGGGCAAGTT-3′	

**Table 2:** The designed primers of the study.

Gene	Primer Sequence	Size (bp)
B_2_m	Forward: 5′-CGTGCTTGCCATTCAGAAA-3′	244
	Reverse: 5′-ATATACATCGGTCTCGGTGG-3′	
Bax	Forward: 5′-CTGCAGAGGATGATTGCTGA-3′	174
	Reverse: 5′-GATCAGCTCGGGCACTTTAG-3′	
Bcl-2	Forward: 5′-ATCGCTCTGTGGATGACTGAGTAC-3′	134
	Reverse: 5′-AGAGACAGCCAGGAGAAATCAAAC-3′	

## Data Availability

The datasets analyzed during the current study are available from the corresponding author on reasonable request.

## Abbreviations

Mesenchymal stem cells (MSCs), Dental pulp stem cells (DPSCs), magnetic resonance imaging (MRI), Phosphate-buffered saline (PBS), Dulbecco’s Modified Eagle’s Medium F12 (DMEM-F12), Fetal bovine serum (FBS), Double distilled water (DDW), Reverse transcriptase–polymerase chain reaction (RT–PCR), Population doubling time (PDT), The 3-4,5-dimethylthiazol-2-yl)-2,5-diphenyl-2H-tetrazolium bromide (MTT) assay, T2*-weighted imaging (T2*WI), Beta-2-Microglobulin (B2m), Fluorescence-Activated Cell Sorting (FACS), National Institute for Medical Research Development (NIMAD).
